# Designing healthcare for human use: Human factors and practical considerations for the translational process

**DOI:** 10.3389/frhs.2022.981450

**Published:** 2023-01-06

**Authors:** G. Franklin Edwards III, Vivian Zagarese, Stephanie Tulk Jesso, Matthew Jesso, Samantha M. Harden, Sarah Henrickson Parker

**Affiliations:** ^1^ Graduate Program in Translational Biology, Medicine and Health, Virginia Tech, Blacksburg, VA, United States; ^2^ Department of Psychology, Virginia Tech, Blacksburg, VA, United States; ^3^ Systems Science and Industrial Engineering, SUNY Binghamton University, Vestal, NY, United States; ^4^ Human Factors, Carilion Clinic, Roanoke, VA, United States; ^5^ Department of Human Nutrition, Foods, and Exercise, Virginia Tech, Blacksburg, VA, United States; ^6^ Department of Health Systems and Implementation Science, Virginia Tech Carilion School of Medicine, Roanoke, VA, United States; ^7^ Fralin Biomedical Research Institute at VTC, Roanoke, VA, United States

**Keywords:** adoption, contextual factors, face shield, infection prevention, maintenance, re-aim, work system, adaptations

## Abstract

In recent years, the focus of implementation science (IS) shifted to emphasize the influence of contextual factors on intervention adaptations in clinical, community, and corporate settings. Each of these settings represent a unique work system with varying contexts that influence human capabilities, needs, and performance (otherwise known as “human factors”). The ease of human interaction with a work system or an intervention is imperative to IS outcomes, particularly adoption, implementation, and maintenance. Both scientific approaches consider the “big picture” when designing interventions for users and stakeholders to improve work and health outcomes. IS and human factors are therefore complementary in nature. In this paper, the authors will (1) provide perspective on the synergistic relationship between human factors and IS using two illustrative and applied cases and (2) outline practical considerations for human factors-based strategies to identify contextual factors that influence intervention adoption, implementation, and maintenance dimensions of the RE-AIM framework. This article expands on recent research that developed user- and human-centered design strategies for IS scientists to use. However, defining the complementary relationship between IS and human factors is a necessary and valuable step in maximizing the effectiveness of IS to transform healthcare. While IS can complement practitioners' identification of intervention adaptations, human interaction is a process in the work system often overlooked throughout implementation. Further work is needed to address the influence that organizational endorsement and trust have on intervention adaptations and their translation into the work system.

## Introduction

Billions of tax dollars are spent on health services research annually (1), but the adoption and maintenance of evidence-based interventions lag (2). Issues with the uptake of an intervention are noted throughout the history of medicine (3). Given this lag in translation and uptake, solely determining the effectiveness of a clinical innovation is not sufficient to ensure its routine use but addressing the challenges of implementing an intervention could bridge this gap (3). In the late 1990s and early 2000s, implementation science (IS) developed as a discipline with methods for testing the integration of interventions in practice settings (4). While the evidence of the effectiveness of an intervention does contribute to some adoption rates, mediating contextual issues, such as different disciplines, appropriate outcomes, and usability of the intervention itself, are also highly influential (5).

According to the International Ergonomics Association, human factors developed during the 1940s and is a “scientific discipline concerned with the understanding of interactions among humans and other elements of a system, and the profession that applies theory, principles, data, and methods to design in order to optimize human well-being and overall system performance” (6). Typically, the study of human factors takes place in high-risk work settings, such as aviation or nuclear power; however, over the last two decades, human factors have become further integrated into the delivery of healthcare to reduce errors and improve efficiency (7). Simply stated, the basic tenets of human factors are: (1) that the system influences how individuals interact within it, (2) that there are experimentally tested and consistent findings demonstrating that humans have inherent capabilities and limitations, and (3) that the design of a work system can account for these capabilities and limitations to support human performance (8).

The tenets of human factors complement those of IS and could further facilitate practical applications for IS interventions (9). IS has methods to understand the intervention, the implementation strategy, and the outcomes of interest (10). A large body of literature, ranging from human-computer interactions to mental health services, suggests the human-centeredness of interventions and the design of the work system also heavily influence translation (11–14). If humans do not perform to an expected standard, it is likely that the failure stems from a mismatch between the system and human capabilities to function within that system (15–18).

It is possible that gaps in translation, specifically implementation, adoption, and maintenance, are related to the discordance between the intervention of interest, the design of the “work” or intervention of interest, implementation strategy, and human capabilities (19). Applying this perspective to IS in both health and healthcare, the human-centeredness of the design of interventions (based on understanding the capabilities of the end users) could heavily influence their translation (11, 12). The aim of this paper is to use part of the reach, effectiveness, adoption, implementation, and maintenance (RE-AIM) framework to outline where human factors strategies can complement the translational process (20–23). Specifically, this paper will focus on applications of human factors methodology and principles to improve adoption, implementation, and maintenance activities. To this end, the authors leverage the recent work of members of the authorship team (MJ, SHP) in infection prevention for illustrative purposes.

Based on findings from the field of human factors and practical considerations for the dimensions of the RE-AIM framework, the authors suggest an opportunity for integrating methods where applicable in the translational process. In this article, authors strategically chose two case studies to elucidate the interactivity of human factors and IS: (1) the complexity of hand sanitizing practices in the context of outpatient dialysis and (2) how human factors practices contributed to the development of ergonomic personal protective equipment (PPE) (face shields) during the COVID-19 pandemic response. Each case study offers a complex IS issue, explored through the lens of human factors. Although both cases are related to infection prevention the goal is to provide insight beyond this issue, to better understanding how implementation is impacted by work system design. In the discussion, the authors will categorize human factors strategies, according to the adoption, implementation, and maintenance dimensions of the RE-AIM framework.

### Case study #1: considering human factors in the design of hand sanitizing stations in outpatient dialysis clinics

Patients with chronic kidney disease are particularly vulnerable to infections, and infections are the second leading cause of death in outpatient dialysis patients (24, 25). When receiving dialysis in an outpatient facility, patients are susceptible to infection because their personal dialysis connection site (either catheter or fistula/graft) is exposed during their dialysis procedure. Interventions such as appropriate hand hygiene and specific wound cleaning techniques have been established as a protection against infection (26). However, infections in these settings remain, even though a best practice for prevention is well established (26).

Members of the authorship team (MJ, SHP) used a macroergonomic approach in inpatient dialysis to better understand the human factors contributions to non-adherence to infection prevention evidence. For the purposes of this article, the authors will focus on one evidence-based best practice, hand sanitizing, and how the design of the system impacted implementation. An in-depth description of the overall methods and analysis can be found elsewhere (27). The Systems Engineering Initiative for Patient Safety (SEIPS) model was used to identify “work system inputs, care processes, and their influence on outcomes related to the patient and provider” in inpatient dialysis clinics (28, 29). The study identified factors related to the individual providers (e.g., the impact of disruptions on cognitive processes), the physical layout of the space, the technology and tools, and the organization (e.g., scheduling) were related to whether hand sanitizing was implemented properly.

There was wide variation in facility layout, with focus group feedback indicating that the location of hand sanitizing stations was often inconvenient for the work processes. In observations, individuals would go outside of normal walking paths to use hand sanitizing stations. Interruptions and alarms were frequent, occurring at a rate of 19% and 50.6% of all patient encounters respectively. Interruptions frequently resulted in additional hand sanitizing needs, as they were often caused by the machine and had to be silenced by physically touching an unclean surface. These findings illustrate how the design of the work system is impacting the action of engaging in a best practice (i.e., hand sanitizing).

Patient scheduling was observed to be rigidly timed, and limited flexibility in turnover time was observed at multiple centers. Although intervening events such as patient transportation delays, treatment interruptions for patients' needs, difficulty with needle insertion or delayed clotting were routinely observed, extra time was not routinely allotted for such occurrences. Dialysis connection and disconnection activities often overlapped. While there is not a direct correlation to hand hygiene, the context of the work is influencing how individuals engage with hand sanitizing stations.

This is an example of how successful implementation of an established intervention (i.e., hand sanitizing to prevent infection) is entangled with the design of the work system, and the human factors of the individual workers (e.g., forgetting or making mistakes when rushing or multitasking) within the work system. Designing the implementation of hand sanitizing stations to match the human capabilities and limitations may help facilitate adoption.

### Case study #2: inclusion of human factors in the design and implementation of face shields during COVID-19

During the COVID-19 pandemic, healthcare supply chains experienced a shortage of medical equipment. An Emergency Use Authorization allowed frontline staff to wear improvised PPE to protect themselves from contact with bodily fluids during patient care (30). In the early phases of the pandemic, while there was significant concern over the contagious nature of SarsCoV-2 (the virus that causes COVID-19), face shields were recommended (31, 32).

The benefits of properly wearing face shields are well-known (33); however, uncomfortable, or poorly designed equipment can lead to staff non-compliance. This issue is critical to resolve for both staff safety and to help limit cross contamination. To better understand why an individual might be non-compliant with face shielding best practices, members of the authorship team (MJ, SHP) conducted an iterative design process with frontline providers and human factors experts. By contrast to the previously presented case study, this study was focused only on individuals and the context for face shielding, not on a broad macroergonomic perspective. MJ, SHP, and others attempted to use human factors principles, particularly around hardware usability, and user feedback to drive the study.

Key elements for design were identified from 1,648 survey responses such as the ability to adjust tension, anti-fogging, ventilation, and durability (34). A 3-phase iterative, randomized trial was then conducted with frontline providers. To measure the success of the design iterations, Kurtz et al. (2022) conducted a repeat survey after each phase, identifying common issues. The final design was able to meet the design criteria and limit or eliminate the common issues (see Figure 1 in reference 34).

This is an example of how the implementation of a best practice that benefits frontline providers as well as patients, can potentially be augmented by good design at the individual level. Where the previous example examined the broader work system contributions to implementation, this example illustrates individual requirements for implementing an evidence-based practice (i.e., wearing a face shield to prevent infection).

## Discussion

These two case studies illustrate the benefit of integrating human factors principles into intervention design and are applicable to the implementation of other evidence-based practices. Identifying linkages between the principles, tools, and methods of both human factors and IS may improve adoption, implementation, and maintenance of evidence-based practices like hand sanitizing in outpatient dialysis clinics and wearing face shields in hospital departments. Notably, the authorship team has expertise in human factors and IS, but do not claim to be experts in infection prevention. In future investigations, the goal is to use both disciplines to maximize human-centeredness and uptake of interventions, given the interdependent contextual factors and human capabilities (21, 23).

To further illustrate the associations between human factors and IS, the authors use the three dimensions of the RE-AIM framework that are associated with IS outcomes of staff and setting levels: adoption, implementation, and maintenance; then further articulate the human factors considerations for each construct with the two exemplar case studies (Table 1).

**Table 1:** Adoption, implementation and maintenance and human factors considerations.

Implementation science construct	Human factors considerations	Exemplar case study specific considerations and *explanations*
**Adoption** - “the absolute number, proportion, and representativeness of settings and intervention agents (i.e., people who deliver the program) who are willing to initiate a program” (Glasgow et al., 1999)	1. Does the design of the intervention align with human capabilities (e.g., physical, cognitive)? 2. Does the design of the intervention avoid overtaxing cognitive or physical resources? 3. How is technology utilized in the design? 4. How are individuals trained to complete the steps in an intervention? 5. In what ways does the intervention fit within the user's current work and workflow? In what ways does the intervention hinder the current work and workflow?	(Case study #1) Does the feel, smell, and time-to-dry of the hand sanitizer affect usage? Explanation: *If the sanitizer does not dry fast enough, users may avoid it or not use it properly because it needs to be dry to do their next task*. (Case study #2) Did people wear the face shields for the entire shift? Explanation: *If the design makes individuals’ heads hurt or makes it difficult for them to see, they will not use the intervention, even if they are asked to by management.*
**Implementation (setting level)** - “the intervention agents’ fidelity to the various elements of an intervention's protocol, including consistency of delivery as intended and the time and cost of the intervention” (Glasgow et al., 1999)	1. What is the context and motivational factors? 2. Can the end users use the intervention consistently? 3. Does work as performed allow fidelity? 4. Is there a physical or mental cost of intervention? 5. What is the work system/workflow?	(Case study #1) Is the sanitizing station easy to access (e.g., near the patient's station, visible, unobstructed, ergonomic access)? Explanation: *If it is difficult to physically access the sanitizing station, individuals are less likely to use it, or use it improperly even though evidence supports its usage*. (Case study #2) Does the face shield affect donning and doffing during different use scenarios (e.g., emergency vs. normal patient care)? Explanation: *Despite its protectiveness against germ spread, if donning the face shield is difficult or requires too many steps, it will not be utilized, even if the organization is bought in. The implementation fidelity is impacted by the design.*
**Maintenance (setting level)** - “the extent to which a program or policy becomes institutionalized or part of the routine organizational practices and policies” (Glasgow et al., 1999)	1. How easy is it to understand what to do? 2. Does training have to be redone for users to be able to do the intervention? 3. What kind of feedback (structure and content and frequency) is given to individuals that they are doing the right thing? 4. How are adaptations considered, implemented, and evaluated? 5. What are other contributing factors to improvement? (setting level) And how do they impact the intervention? 6. What is the cognitive or physical incentive to continue to do the intervention?	(Case study #1) Do healthcare providers stop using hand sanitizing stations during seasonal changes (e.g., dry, and cracked hands in the winter exacerbated by hand sanitizer)? Explanation: *If additional contextual factors (e.g., seasonal changes) are unaccounted for, then hand sanitizing stations could be underutilized*. (Case study #2) Do the face shields continue to meet evolving requirements of frontline clinical work? Is the functionality easily adapted for different use cases? Explanation: *Maintaining an intervention requires behavioral change. If the behavioral change is not supported organizationally, or is difficult or physically uncomfortable, it is not likely that the intervention behaviors will be maintained.*

This work is a novel contribution to the IS literature and expands on recent efforts to identify human-centered implementation strategies (11, 35–37). Researchers and practitioners may find some strategies more helpful than others including task analysis, co-creation sessions, workflow analysis, and iterative prototyping (11, 35, 36). The design of an intervention, including the structure of delivery, hardware or software that can facilitate delivery, and training for target populations (care providers and patients/clients), is highly influential to *how* and *whether* and *for how long* the interventions are adopted and whether the setting's infrastructure can maintain them. Ultimately, the design of both the intervention and the work system are very likely to impact translation as well as implementation of an intervention.

The case studies have integrated both observations of normal work (i.e., hand sanitizing) to develop interventions, and a near real-time iterative process for integrating feedback (i.e., designing a face shield). Documentation of the iterative process is helpful to others that are attempting to implement an intervention in their own setting. From some of the authors' experience (STJ, MJ, SHP), the method of gathering iterative feedback is domain agnostic and ultimately translatable to other clinical settings, whereas the exact findings are contextual and often highly local. Information gathered in real-time informs the perspective of work as performed realistically, providing insight into how an intervention might be integrated. Questions relevant to adoption, (e.g., does the design of the intervention align with human capabilities? How do you avoid overtaxing physical or cognitive resources? Table 1), can address contextual factors that would limit adoption of interventions like hand sanitizing stations and face shields in high-risk work environments. For example, the real-time information gathering revealed that a hand sanitizer which does not dry quickly or has an uncomfortable texture (e.g., gritty, slippery, etc.) may limit uptake. Similarly, face shield uptake is dependent on the ability of the device to fit seamlessly within normal work duties and certainly cannot cause physical discomfort and should not make performing job functions more difficult.

Contextual factors influence every aspect of implementation. Systematically identifying contextual issues, including human performance issues, results in a better intervention design, and a higher likelihood of uptake (11, 35, 36, 38, 39). The field of human factors has much to offer on this point. To facilitate use in IS, the authors developed a list of implementation questions (Table 1). In these examples, the relevant implementation questions are centered around the consistent use of the intervention and whether work as performed allows for fidelity to the intervention. For example, an important contextual issue to consider while implementing the sanitizing stations is whether they are easy to access (e.g., accessible, visible, unobstructed, etc.). Logically, if a sanitizing station is difficult to access, then individuals may be less likely to use it. This conceptualization extends to donning and doffing of face shields: does the poor design of face shields interfere with consistent use of the intervention? If so, the design of an intuitive and ergonomic face shield becomes imperative because an individual is less likely to wear a face shield if it is cumbersome and poorly designed.

Stakeholders are critical at all phases of both human factors and IS (39–41). In human factors, the primary stakeholders of concern are frontline users of a product, device, or process. Including the frontline users in research discussions elevates the feedback from the individuals who know what may or may not be successful for their daily work. Frontline workers, otherwise, the end-users, can help researchers and implementation science practitioners understand the workflow in a particular setting. For example, including frontline users in the layout of hand sanitizing stations could possibly increase uptake and buy-in. A frontline user can identify the (1) critical times hand sanitizing must be performed, (2) paths in the workflow that do not burden task completion, and (3) accessible locations. This idea extends beyond hand sanitizing and face shield donning and doffing but can be used to identify promising strategies for implementing an intervention at multiple points of human interaction in a work system. In IS, stakeholders are also often organizational leaders or other individuals. While these individuals are critical to the resources needed to develop and implement an intervention, they are not the actual “doers” of the activity, and therefore, may have complementary insight into the daily work of frontline staff. A dichotomy stating that human factors consider frontline users and IS does not or IS considers organizational stakeholders and human factors does not, would be false. Each discipline is beneficial to the other in terms of designing for implementation success.

## Conclusion

In this paper, the authors suggest that incorporating strategies from a human factor's perspective is a minor but pivotal shift within an implementation study. As illustrated in the two applied case studies, infection prevention in outpatient dialysis clinics and hospital departments that require face shields is more likely to succeed if human interactions within a work system are carefully considered in the design and implementation process. The authors attempted to articulate the linkages between IS and human factors and organize strategies according to the interdependent dimensions of the RE-AIM framework. The authors used two case studies as examples that required both an IS mindset and human factors design principles to promote adoption, implementation, and maintenance. It is the authors' belief that both disciplines are complementary of the other, and by integrating principles from each, the implementation of evidence-based practices like hand sanitizing and face shielding are strengthened.

In the field of IS, it is prudent to design an intervention with considerations for its use in the work system. Additionally, including insights from other fields, such as industrial and organizational psychology may prove useful when examining the interactions across levels of organizations. For example, the satisfaction and motivation of employees, and the extent to which the leadership endorses the intervention may affect its adoption and maintenance within the organization.

As demonstrated by the two applied case studies, the consideration of human factors complements the implementation process and likely improves use of an intervention. Consequently, the authors believe that the identification of human factors in an implementation study could substantially improve adoption, implementation, and maintenance.

## Data Availability

The original contributions presented in the study are included in the article, further inquiries can be directed to the corresponding author/s.
